# Precision Motion Control of a Linear Permanent Magnet Synchronous Machine Based on Linear Optical-Ruler Sensor and Hall Sensor

**DOI:** 10.3390/s18103345

**Published:** 2018-10-07

**Authors:** Chih-Hong Lin

**Affiliations:** Department of Electrical Engineering, National United University, Miaoli 36063, Taiwan; jhlin@nuu.edu.tw; Tel.: +886-3738-2464

**Keywords:** backstepping control, Elman neural network, linear permanent magnet synchronous machine, Lyapunov stability

## Abstract

The linear optical-ruler sensor with 1 μm precision mounted in the linear permanent magnet synchronous machine (LPMSM) is used for measuring the mover position of LPMSM in order to enhance the precision of a measured mover position. Due to nonlinear friction and uncertainty effects, linear controllers are very hard to achieve good mover positioning of LPMSM. The proposed adaptive amended Elman neural network backstepping (AAENNB) control system is adopted for controlling the LPMSM drive system to bring about the mover positioning precision of LPMSM. Firstly, a backstepping scheme is posed for controlling the tracing motion of the LPMSM drive system. The proposed backstepping control system, which is applied in the mover position of the LPMSM drive system, possesses better dynamic control performance and robustness to uncertainties for the tracing trajectories. Because of the LPMSM with nonlinear and time-varying dynamic characteristics, an adaptive amended Elman neural network uncertainty observer (AAENNUO) is posed to estimate the required lumped uncertainty. According to the Lyapunov stability theorem, on-line parameter training methodology of the amended Elman neural network (AENN) can be derived by use of adaptive law. The error estimated law is proposed to compensate for the observed error induced by the AENN with adaptive law. Furthermore, to help improve convergence and to obtain better learning performance, the mended particle swarm optimization (PSO) algorithm is utilized for adjusting the varied learning rate of the weights in the AENN. At last, these experimental results, which show better performance, are verified by the proposed control system.

## 1. Introduction

Compared with other classes of linear actuators, most linear machines have smaller load capacity. Linear machines have a merit in outdoor or dirty environments because the stator and mover parts need not contact each other. Moreover, the drive coils can be watertight and sealed against corrosion and moisture and can thus have a long serving life. The linear permanent magnet synchronous machine (LPMSM) which is the direct-drive mechanical design, has lots of merits over the indirect-drive transcript, for instance, no backlash, low friction, high speed, high precision in long distance position, simple structure, and high thrust force [1]. Therefore, the LPMSM is suitable for high precision servo applications, and it has been applied for manufacturing systems and machine tools [1,2,3].

The backstepping design is suitable for a large class of state feedback linearizable systems with nonlinear properties. Its method [4,5,6] is to select certain fit functions of state variables, and to set as pseudo-control inputs in some subsystems of the whole system. Each backstepping section leads to the novel pseudo-control design, and represents another pseudo-control design from previous design steps. When the order is achieved, true control inputs result in the feedback design. By summing up the Lyapunov functions from a virtue of a final Lyapunov function related with individual design step, the original design objective was achieved [4,5,6,7]. However, some methods use a linear model of the machine, which may not be suitable for high-performance applications under the occurrence of uncertainties. On the other hand, the related control methods with a focus on some nonlinear and linear controllers were proposed [8,9,10,11]. Lu et al. [8] posed the control method of cell divisions in the nervous system as symmetry and asymmetry. Precup et al. [9] developed the new Takagi-Sugeno proportional-integral-fuzzy controllers (PI-FCs) to control a class of servo systems with maximum sensitivity. Martin et al. [10] proposed a genetic algorithm for optimal tuning of a networked linear controller, and then applied it to a complex electromechanical process. Vrkalovic et al. [11] developed the design of Takagi-Sugeno fuzzy controllers in state feedback form by the use of swarm intelligence optimization algorithms. The adaptive backstepping controllers were applied in linear induction machine control [12,13]. Furthermore, the adaptive backstepping controllers combined with the neural networks to control the nonlinear systems have been proposed in References [14,15]. However, the error compensation mechanisms in these methods have never been proposed. Thus, the motivation of the proposed adaptive, amended Elman neural network backstepping (AAENNB) control system by use of a linear optical-ruler sensor with 1 μm precision and three Hall sensors provides an error compensation mechanism to enhance the robustness of the system under parameter variations and external force disturbances to raise the control precision. 

Elman [16] proposed an especially partial recurrent neural network (RNN) which is called the Elman neural network (ENN). The classical ENN is a RNN with delayed feedback in the hidden layer. It can provide the standard state-space expressions for some dynamic systems. It can also be regarded as a special kind of RNN with feedback links from the hidden layer to the context layer. The context layer is an additional layer which can be regarded as an extra memory to memorize previous activations of the hidden neurons. It feeds to all the hidden neurons after the one-step time delay. Compared to the ordinary RNNs, the ENN has an unusual strict memory to deposit the provisional information. In general, the ENN can be regarded as an especial kind of feed-forward NN with added memory neurons. The ENN has certain dynamic fits over the static neural network (NN) [17,18,19], owing to the context nodes in the ENN. In addition, the ENN has been applied abroad for identification and control of systems [20,21,22,23]. Furthermore, in order to raise fast convergent capacity and to enhance the high precision ability for high-order nonlinear systems, the amended Elman neural network (AENN) is proposed in this paper.

Owing to structural merit [24,25,26,27], the RNN has become one of most popular NNs in dynamic control and nonlinear modeling. The main property of the RNN is a self-linked to memorize a feedback message of the historical effect in the same node. Moreover, the specific self-link feedback of the hidden node or the output node in the general RNN is in charge of memorizing the particular frontal activation of the hidden node or the output node, and then feeds to itself only. Thus, the outputs of the other nodes have no capacity to involve the particular neuron. However, the friction force and the external force obstruction in the LPMSM with complicated nonlinear dynamic behavior is always serviced as an important term. Hence, if each node in the RNN is regarded as a state in the nonlinear dynamic system, the self-link feedback class cannot approximate the nonlinear dynamic effectively. Moreover, the feedback signals not only are self-linked but also feed to all the hidden nodes in the context nodes of ENN. Thus, the construction of ENN is more formidable than the general RNN for dealing with time-varying and nonlinear dynamic systems because the ENN has more approximated capacity effectively with the extra context layer. Furthermore, some amended Elman neural networks (AENNs) [28,29,30] have been posed recently to improve the ability of identifying high nonlinear systems. It has been proven to have more merits than the basic ENN, such as higher accuracy, better performance, faster transient response and better dynamic robustness.

Due to easy implementation and having a quick convergent capacity for solution optimization, a particle swarm optimization (PSO) has become one of the most popular optimization methods. The best solution in the global region can be found by simply regulating each individual towards its own best position trajectory and toward the best solution at each step [31,32]. In optimization methods, the fit control of global and local exploitations is related to find the optimization solution effectively [33]. Moreover, the PSO has been widely used in multi-sensor data fusion and dynamic model updating for bridge structures [34,35] due to its simple structure, simple parameter setting and fast convergent speed. Furthermore, the PSO has the precocious convergent problem. The mended PSO [36,37] with the fast convergent speed is thus posed to prevent precocious convergence and to obtain an optimized learning rate.

The modeling errors and disturbances can occur in a function that has both unknown parameters and dynamics of the system in the LPMSM drive system. Due to the effect of these uncertainties, the better control performance of the LPMSM drive system is hard to achieve by use of linear controller. In order to raise robustness, an adaptive amended Elman neural network backstepping (AAENNB) control system is posed for controlling the motion of the mover position of the LPMSM drive system to track periodic references. The motion of the mover position of the LPMSM drive by use of the backstepping control system holds the merits of good transient control performance and good robustness under uncertainty interference for two kinds of tracing periodic references. Additionally, to further enhance the robustness of the LPMSM drive, an adaptive amended Elman neural network uncertainty observer (AAENNUO) is posed for estimating the necessary lumped uncertainty. Furthermore, to help improve convergence and to acquire better learning performance, the mended PSO algorithm is utilized for adjusting the varied learning rate of the weights in the AENN. Thus, in this paper, the objective of the AAENNB control system for controlling the LPMSM drive system by use of linear optical-ruler sensor with 1 μm precision and three Hall sensors is to raise the control precision and to enhance the robustness of the system under parameter variations and external force disturbances. The AENN with adaptive law is posed to adapt the lumped uncertainty value. The error estimated law is posed for compensating the observed error that prevailed by the AENN with adaptive law, and we cannot build the convergence of the tracing error to zero. Therefore, all errors defined on the AAENNB control system converge into a neighborhood with reachable radius, and stay within uniformly final boundedness. Moreover, to help improve convergence and to acquire better learning performance, the mended PSO algorithm is utilized for adjusting the varied learning rate of the weights in the AENN. Finally, the robustness and effectiveness of the posed AAENNB control system are shown in some experimental results.

The remainder of this paper is organized as follows: The materials and methods including composition of LPMSM drive and control methods are reviewed in Section 2. Experimental results and discussion including some experimental results and characteristic performance comparisons for the three control systems are presented in Section 3. Some conclusions are given in Section 4.

## 2. Materials and Methods

### 2.1. Composition of LPMSM Drive

The *d-q* axis voltage model of the LPMSM can be presented in synchronous rotating reference frame in References [1,2,3]) as follows:

(1) $$v_{q}=R_{s}i_{q}+L_{q}{\dot{i}}_{q}+\omega_{e}(L_{d}i_{d}+\lambda_{m})$$

(2) $$v_{d}=R_{s}i_{d}+(L_{d}{\dot{i}}_{d}+{\dot{\lambda }}_{m})-\omega_{e}L_{q}i_{q}$$

where $\omega_{r}$ and $\omega_{e}=P\omega_{r}/2$ are the electrical angular speeds of the mover and switching power. $v_{d}$, $i_{d}$ and $L_{d}$ are the *d* axis voltage, current and inductance, respectively. $v_{q}$, $i_{q}$ and $L_{q}$ are the *q* axis voltage, current and inductance, respectively. $\lambda_{m}$, $R_{s}$ and P are the flux linkage of permanent magnet, the phase winding resistance and the number of pole, respectively. Furthermore,

(3) $$\omega_{r}=\pi u_{r}/n_{r}$$

(4) $$u_{e}=Pu_{r}/2=\tau f_{t}$$

where $u_{e}$, $u_{r}$, $n_{r}$ and $f_{t}$ are the electrical linear speed, the linear speed, the pole pitch and the electrical frequency, respectively. The electromagnetic force [2] can be denoted by

(5) $$f_{d}=3\pi P[\lambda_{m}i_{q}+\left(L_{d}-L_{q}\right)i_{d}i_{q}]/(4n_{r})$$

The electromagnetic power [2] can be denoted by

(6) $$p_{d}=f_{d}u_{e}=3P[\lambda_{m}i_{q}+\left(L_{d}-L_{q}\right)i_{d}i_{q}]\omega_{e}/4$$

The dynamic equation of the mover can be denoted as

(7) $$f_{d}=M{\dot{u}}_{r}+Du_{r}+f_{l}$$

where $f_{l}$, $f_{d}$, *M* and *D* are the external force disturbance, the electromagnetic force, the total mass of the moving element system and the viscous friction, respectively.

The control method based on field orientation mechanism [2] for the LPMSM drive system is adopted. The flux linkage position in the *d-q* axis frame can be determined by three Hall sensors. In (5) and (6), if $i_{d}=0$ and $\lambda_{m}$ is constant for the LPMSM, then the electromagnetic force $f_{d}$ is proportional to $i_{q}$, which is determined by closed-loop control. The rotor flux linkage is produced by the *d*-axis current only, and the force current is generated by the *q* axis current for the field-oriented control. Since the generated machine force is linearly proportional to the *q* axis current when the *d* axis rotor flux linkage is constant in (5), the maximum force per ampere can be achieved. The simplified force equation is given by

(8) $$f_{d}=3\pi \lambda_{m}i_{q}/(4n_{r})$$

The optimized electromagnetic performance for the LPMSM drive system is thus implemented by controlling the main current distribution to lie in the *q* axis, i.e., $i_{d}=0$, and this will yield a linear force per amp characteristic for the LPMSM drive system.

**Figure 1:**
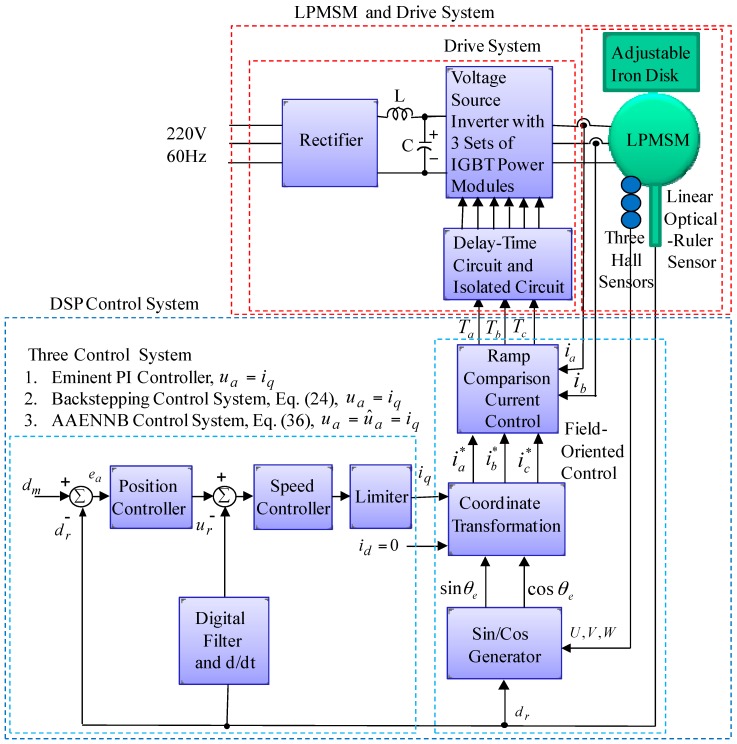
Formation of LPMSM drive system with field-oriented control using DSP control system.

The formation of the LPMSM drive system with field-oriented control is shown in Figure 1, which is comprised of a LPMSM, a ramp comparison current control, a coordinate transformation, a cos/sin generator, a speed control, a position control, a linear optical ruler sensor and three Hall sensors. A linear optical ruler sensor with 1 μm precision is used for detecting the motion position of the mover. The linear optical ruler sensor is a DC 5V grating ruler. A linear optical ruler sensor has three digital output signals, A/B/Z and $\bar{A}/\bar{B}/\bar{Z}$, which issue quadrature squarewaves and zero pulse. Depending on its internal mechanism, an encoder may derive *A* and *B* directly from sensors which are fundamentally digital signals in nature, or it may interpolate its internal signals. Then, the four-multiplier converter circuit converts 1000 pulses into 1 mm = 0.5 V. In this case, the interpolation process effectively sub-divides the scale period and thereby achieves higher measurement resolution. Moreover, three Hall sensors denoted *U*, *V* and *W* are used to detect the flux linkage position of the permanent magnet (PM). Three Hall sensors consist of the Hall elements and the associated electronics which is a basic analog output device. Analog sensors provide an output voltage that is proportional to the magnetic field to which it is exposed. The additional circuit functions were added to simplify the application. The analog sensor accepts a 4.5 V to 10.5 V supply voltage. The sensor has a sensitivity (mV/Gauss) and offset proportional (ratiometric) to the supply voltage. This device has rail-to-rail operation. Its output varies from almost zero (0.2 V typical) to almost the supply voltage (*Vs*, 0.2 V typical). The basic analog output device can be converted into a digital output sensor with the addition of a Schmitt trigger circuit.

The iron disks with different sizes and spring with the same or the opposite force can be mounted on the mover of LPMSM to change the mass of the moving element and viscous friction, i.e., the parameters disturbance with four times the nominal value of the mover mass and viscous friction. 

**Figure 2:**
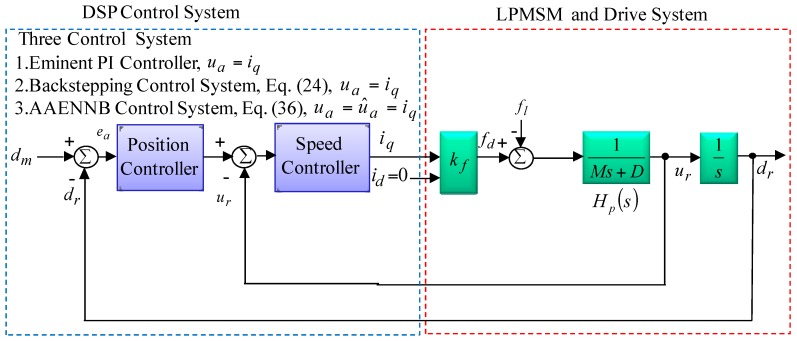
Simplified block diagram of the LPMSM drive and control system.

The field-oriented control was implemented by use of digital-signal-processor (DSP) TMS320C32 control system. The LPMSM drive and control system by use of field-oriented control in References [1,2,3] can be simplified as shown in Figure 2, where

(9) $$f_{d}=k_{f}i_{q}$$

(10) $$k_{f}=3\pi P\lambda_{m}/(4n_{r})$$

(11) $$H_{p}(s)=1/(Ms+D)$$

where $k_{f}$ is the thrust coefficient. $i_{q}=i_{q}^{*}$ is the command of thrust current. *s* is a Laplace’s operator. The specifications of LPMSM with linear optical ruler sensor with 1 μm precision in this study are given as 220 V, 3.1 A, 0.6 kW, 50.8 N. The position and speed signals in the control loop are set at 1 V = 2 mm and 1 V = 2 mm/s for the convenience of the controller design. The parameters of the system are given as $\bar{M}=2.1\;\mathrm{kg}=0.1812\mathrm{Ns}/V$, $\bar{D}=81.62\;\mathrm{kg}/s=5.021\;N/V$, $k_{f}=32.2N/A$. The “−” symbol represents the system parameter in the nominal case. 

### 2.2. Control Methods

By use of (7), the actual LPMSM drive system including parameter variations, external load disturbance, and friction force can be presented by

(12) $${\dot{d}}_{r}=u_{r}=x_{a}$$

(13) $${\dot{x}}_{a}=(a_{1}+\Delta a_{1})x_{a}+(b_{1}+\Delta b_{1})u_{a}+c_{1}f_{l}$$

(14) $$y=d_{r}$$

where $d_{r}$ and $x_{a}$ are the mover position and speed of the LPMSM. $a_{1}=-D/M$, $b_{1}=k_{f}/M>0$ and $c_{1}=-1/M$ are three constants. $\Delta a_{1}$ and $\Delta b_{1}$ are two uncertainties from *M* and *D* parameters variations. $u_{a}=i_{q}^{}$ is the control effort which inputs to the LPMSM drive system, i.e., the thrust current. Then (13) can be denoted by

(15) $${\dot{x}}_{a}=ax_{a}+bu_{a}+l_{a}$$

and

(16) $$l_{a}\equiv \Delta ax_{a}+\Delta bu_{a}+cf_{l}$$

where $l_{a}$ is called the lumped uncertainty. The lumped uncertainty will be inspected by an adaptive uncertainty observer. It is assumed that the observation is a constant. Because the sampling period of the observer is very short compared with the variation of $l_{a}$, the constant assumption is valid in practical digital processing of the observer.

The control aim is that the output y(t) of the system can trace the reference trajectory $y_{d}(t)$, i.e., $d_{m}$, asymptotically. The control system is designed to achieve the position-tracing aim, and is described by use of the backstepping control system as follows.

***Order 1:*** Define the tracing error for the position-tracing aim as

(17) $$e_{a}=d_{m}-d_{r}=y_{d}-y$$

Its derivative is denoted by

(18) $${\dot{e}}_{a}={\dot{y}}_{d}-\dot{y}={\dot{y}}_{d}-x_{a}$$

Define the following stabilizing function:

(19) $$\beta_{a}=c_{a}e_{a}+{\dot{y}}_{d}+c_{b}\sigma$$

where $c_{a}$ and $c_{b}$ are positive constants. $\sigma =\int e_{a}(\tau )d\tau$ is the integral factor. The tracing error can converge to zero by the use of an integral factor. 

Define the virtual tracing error as

(20) $$e_{b}=x_{a}-\beta_{a}$$

The derivative of $e_{b}$ can be presented by

(21) $${\dot{e}}_{b}={\dot{x}}_{a}-{\dot{\beta }}_{a}=(ax_{a}+bu_{a}+l_{a})-{\dot{\beta }}_{a}$$

The lumped uncertainty $l_{a}$ can be assumed to be bounded, i.e., $\left|l_{a}\right|\leq {\bar{l}}_{a}$. In order to design the backstepping control system, the first Lyapunov function is defined by

(22) $$f_{a}=e_{a}^{2}/2+e_{b}^{2}/2+c_{b}\sigma_{}^{2}/2$$

By using (18) and (21), then the derivative of $f_{a}$ can be presented by

(23) $$\begin{array}{l} {\dot{f}}_{a}=e_{a}{\dot{e}}_{a}+e_{b}{\dot{e}}_{b}+c_{b}\sigma \dot{\sigma }\\ =e_{a}({\dot{y}}_{d}-x_{a})+e_{b}^{}(ax_{a}+bu_{a}+l_{a}-{\dot{\beta }}_{a})+c_{b}\sigma \dot{\sigma }\\ =e_{a}(-c_{a}e_{a}-c_{b}\sigma -e_{b})+e_{b}^{}[a(e_{b}+\beta_{a})+bu_{a}+l_{a}-{\dot{\beta }}_{a}]+c_{b}\sigma \dot{\sigma }\\ =-c_{a}e_{a}^{2}-e_{a}e_{b}+e_{b}^{}[a(e_{b}+\beta_{a})+bu_{a}+l_{a}-{\dot{\beta }}_{a}] \end{array}$$

Then the posed backstepping control system $u_{a}=i_{q}$ from (23) is designed as

(24) $$u_{a}=i_{q}=b_{}^{-1}[e_{a}-c_{c}e_{b}-a(e_{b}+\beta_{a})+{\dot{\beta }}_{a}-{\bar{l}}_{a}sgn(e_{b})]$$

where $c_{c}$ is positive constant. Substituting (24) into (23), then (23) can be obtained by

(25) $$\begin{array}{l} {\dot{f}}_{a}=-c_{a}e_{a}{}^{2}-c_{c}e_{2}{}^{2}+e_{b}l_{a}-\left|e_{b}\right|{\bar{l}}_{a}\\ \leq -c_{a}e_{a}{}^{2}-c_{c}e_{b}{}^{2}-\left|e_{b}\right|({\bar{l}}_{a}-\left|l_{a}\right|)\\ \leq -c_{a}e_{a}^{2}-c_{c}e_{b}^{2} \end{array}$$

Define the following term:

(26) $$\varepsilon (t)=c_{a}e_{a}^{2}+c_{b}e_{b}^{2}\leq -{\dot{f}}_{b}$$

Then

(27) $$\int_{0}^{t}\varepsilon (\tau )d\tau \leq f_{b}(e_{a}(0),\;e_{b}(0))-f_{b}(e_{a}(t),e_{b}(t))$$

**Figure 3:**
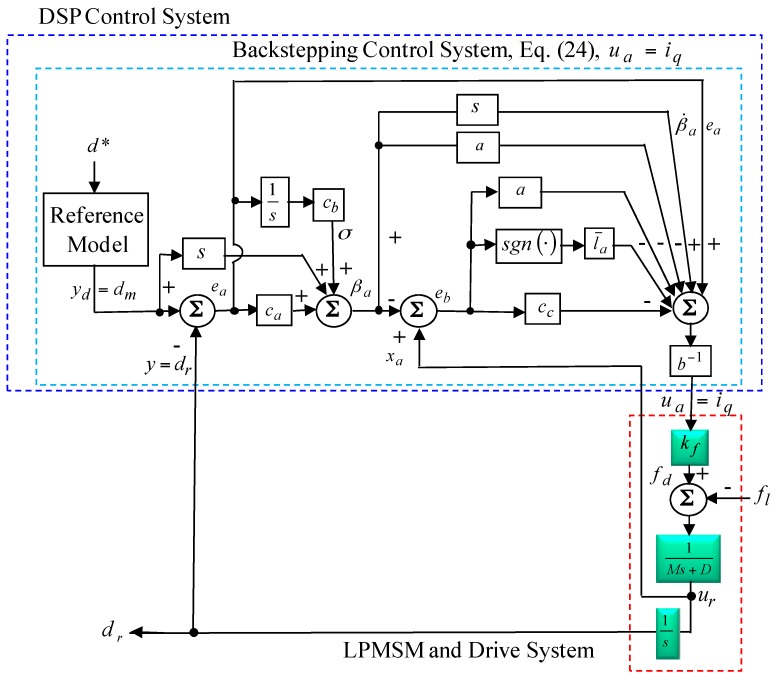
Block diagram of the posed backstepping control system using DSP control system.

Since $f_{a}(e_{a}(0),\;e_{b}(0))$ is limited, and $f_{a}(e_{a}(t),\;e_{b}(t))$ is nonincreasing and limited, then $\underset{t\to \infty }{\lim}\int_{0}^{t}\varepsilon (\tau )d\tau <\infty$. Moreover, $\dot{\varepsilon }(t)$ is limited then and ε(t) is uniformly successive [38,39]. By use of Barbalat’s lemma [38,39], it represents $\underset{t\to \infty }{\lim}\varepsilon (t)=0$, then $e_{a}$ and $e_{b}$ will converge to zero as t→∞. Furthermore, $\underset{t\to \infty }{\lim}y(t)=y_{d}$ and $\underset{t\to \infty }{\lim}x_{a}={\dot{y}}_{d}$. Thus, the posed backstepping control system will be asymptotically stable. The stability of the posed backstepping control system can be guaranteed, and then the block diagram of the posed backstepping control system is shown in Figure 3. 

**Figure 4:**
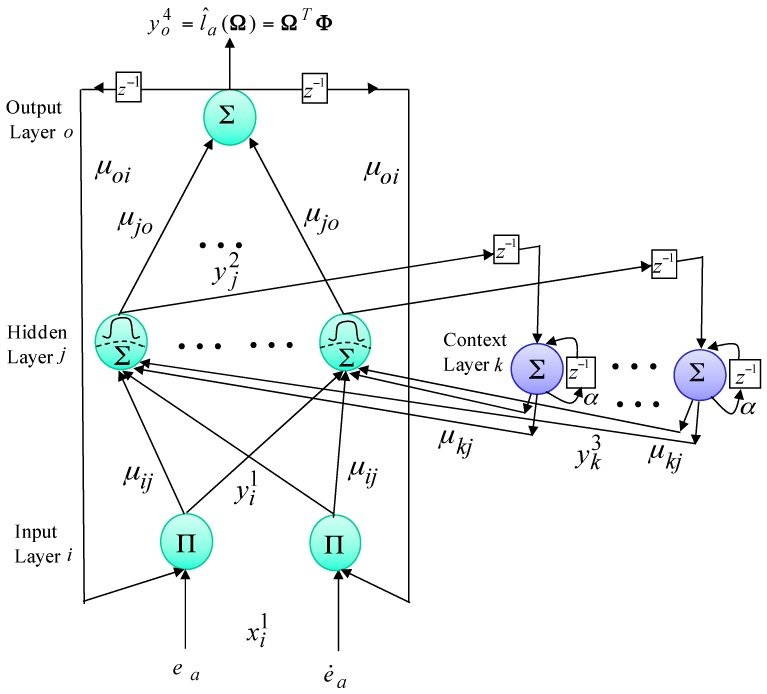
Composition of the four-layer AENN.

***Order 2:*** Because the lumped uncertainty $l_{a}$ is hard to measure and the upper bound ${\bar{l}}_{a}$ is hard to determine, therefore, an AENN uncertainty observer is posed to adapt the value of the lumped uncertainty. The composition of the proposed four-layer AENN, which involves the input, hidden, context and output layers, is shown in Figure 4 to be used to carry out the estimation of the lumped uncertainty. The models in each layer are represented by

***Layer 1: Input Layer i***

(28) $$net_{i}^{1}\left(N\right)=\prod_{o}x_{i}^{1}(N)\mu_{oi}y_{o}^{4}(N-1),\;y_{i}^{1}\left(N\right)=f_{i}^{1}(net_{i}^{1}(N))=net_{i}^{1}(N)),\;i=1,\;2$$

***Layer 2: Hidden Layer j***

(29) $$net_{j}^{2}\left(N\right)=\sum_{k}\mu_{kj}y_{k}^{3}\left(N\right)+\sum_{i}\mu_{ij}y_{i}^{1}\left(N\right),\;y_{j}^{2}\left(N\right)=f_{j}^{2}\left(net_{j}^{2}\left(N\right)\right)=\frac{1}{1+e^{-net_{j}^{2}\left(N\right)}},\;j=1,\;2,\;\ldots ,\;m_{1}$$

***Layer 3: Context Layer k***

(30) $$net_{k}^{3}\left(N\right)=y_{j}^{2}\left(N-1\right)+\alpha y_{k}^{3}\left(N-1\right),\;y_{k}^{3}\left(N\right)=f_{k}^{3}(net_{k}^{3}(N))=net_{k}^{3}(N)),\;k=1,\;\cdots ,\;n_{1}$$

***Layer 4: Output Layer o***

(31) $$net_{o}^{4}\left(N\right)=\sum_{j}\mu_{jo}y_{j}^{2}\left(N\right),\;y_{o}^{4}\left(N\right)=f_{o}^{4}\left(net_{o}^{4}\left(N\right)\right)=net_{o}^{4},\;o=1$$

where $e_{a}$, ${\dot{e}}_{a}$ and $x_{i}^{1}$ are the difference between the reference model $d_{m}$ and the mover position $d_{r}$, the derivative and the *i*th input signal of the input layer, respectively. $m_{1}$, $n_{1}$, *N* and $z^{-1}$ denote the number of neurons in the hidden layer, the number of neurons in the context layer, the number of iterations and the time delay, respectively. $\mu_{ij}$, $\mu_{kj}$ and $\mu_{oi}$ are the connective weights between the input layer and the hidden layer, the connective weight between the context layer and the hidden layer and the recurrent weight between the output layer and the input layer, respectively. $y_{i}^{1}(N)$, $y_{j}^{2}(N)$, $y_{k}^{3}(N)$ and $y_{o}^{4}(N)$ are the *i*th output of the input layer, the *j*th output of the hidden layer, the *k*th output of the context layer and the *o*th output of the output layer, respectively. $f_{i}^{1}$,$f_{j}^{2}$, $f_{k}^{3}$ and $f_{o}^{4}$ are the linear function, the sigmoid function, the linear function and the linear function, respectively. α is the self-link feedback gain of the context layer between 0 and 1. The output $y_{o}^{4}(N)$ of the AENN is represented by

(32) $$y_{o}^{4}(N)={\hat{l}}_{a}(\Omega )=\Omega^{T}\Phi$$

where $\Omega ={\left[\mu_{1o}^{}\;\mu_{2o}^{}\;\cdots \cdots \;\mu_{m_{1}o}^{}\right]}^{T}$ is the vector of the weights of the AENN. $\Phi ={\left[x_{1}^{4}\;x_{2}^{4}\;\cdots \cdots \;x_{m_{1}}^{4}\right]}^{T}$ is the input vector of the output layer, which is determined by the selected sigmoid function and $0\leq x_{j}^{4}\leq 1$.

To exploit the adaptive law of the AENN uncertainty observer, the minimum rebuilt error δ is defined by

(33) $$\delta =l_{a}-l_{a}(\Omega^{*})$$

where $\Omega^{*}$ is an optimized weight vector that reaches the minimum rebuilt error. The |δ| is less than a small positive constant, $\bar{\delta }$, i.e., $\left|\delta \right|\leq \bar{\delta }$. Then, the Lyapunov function is chosen as

(34) $$f_{b}=f_{a}+{\left(\hat{\delta }-\delta \right)}^{2}/(2\gamma )+{\left(\Omega -\Omega^{*}\right)}^{T}(\Omega -\Omega^{*})/\eta_{1}$$

where γ and λ are positive constants. $\hat{\delta }$ is the estimated value of the minimum rebuilt error δ. The estimation of the rebuilt error is used to compensate the observed error induced by the AENN uncertainty observer, and to further guarantee the system stable. Take the derivative of the Lyapunov function from (34)

(35) $${\dot{f}}_{b}={\dot{f}}_{a}+(\hat{\delta }-\delta )\dot{\hat{\delta }}/\gamma +{\left(\Omega -\Omega^{*}\right)}^{T}\dot{\Omega }/\eta_{1}\;=-c_{a}e_{a}^{2}-e_{a}e_{b}+e_{b}^{}[a(e_{b}+\beta_{a})+bu_{a}+l_{a}-{\dot{\beta }}_{a}]+(\hat{\delta }-\delta )\dot{\hat{\delta }}/\gamma +{\left(\Omega -\Omega^{*}\right)}^{T}\dot{\Omega }/\eta_{1}$$

According to (35), an AAENNB control system $u_{a}={\hat{u}}_{a}=i_{q}$ is proposed as follows:

(36) $$u_{a}={\hat{u}}_{a}=i_{q}=b_{}^{-1}[e_{a}-c_{c}e_{b}-a(e_{b}+\beta_{a})+{\dot{\beta }}_{a}-{\hat{l}}_{a}-\hat{\delta }]$$

From (36) and (35), the following equation can be obtained

(37) $${\dot{f}}_{b}=-c_{a}e_{a}{}^{2}-c_{c}e_{b}^{2}+e_{b}l_{a}-e_{b}{\hat{l}}_{a}-e_{b}\hat{\delta }+\frac{(\hat{\delta }-\delta )\dot{\hat{\delta }}}{\gamma }+\frac{{\left(\Omega -\Omega^{*}\right)}^{T}\dot{\Omega }}{\eta_{1}}\;=-c_{a}e_{a}{}^{2}-c_{c}e_{b}^{2}+e_{b}(l_{a}-{\hat{l}}_{a}(\Omega^{*}))-e_{b}({\hat{l}}_{a}(\Omega )-{\hat{l}}_{a}(\Omega^{*}))-e_{b}{\hat{l}}_{a}-e_{b}\hat{\delta }+\frac{(\hat{\delta }-\delta )\dot{\hat{\delta }}}{\gamma }+\frac{{\left(\Omega -\Omega^{*}\right)}^{T}\dot{\Omega }}{\eta_{1}}\;=-c_{a}e_{a}{}^{2}-c_{c}e_{b}^{2}-e_{b}{\left(\Omega -\Omega^{*}\right)}^{T}\Phi -e_{b}{\hat{l}}_{a}-e_{b}(\hat{\delta }-\delta )+\frac{(\hat{\delta }-\delta )\dot{\hat{\delta }}}{\gamma }+\frac{{\left(\Omega -\Omega^{*}\right)}^{T}\dot{\Omega }}{\eta_{1}}$$

The adaptive law for $\dot{\Omega }$ and an error estimated law for $\dot{\hat{\delta }}$ are designed as:

(38) $$\dot{\Omega }=\eta_{1}e_{b}\Phi$$

(39) $$\dot{\hat{\delta }}=\gamma e_{b}$$

Thus, (37) can be rewritten as follows:

(40) $${\dot{f}}_{b}=-c_{a}e_{a}{}^{2}-c_{c}e_{b}^{2}=-\varepsilon (t)\leq 0$$

**Figure 5:**
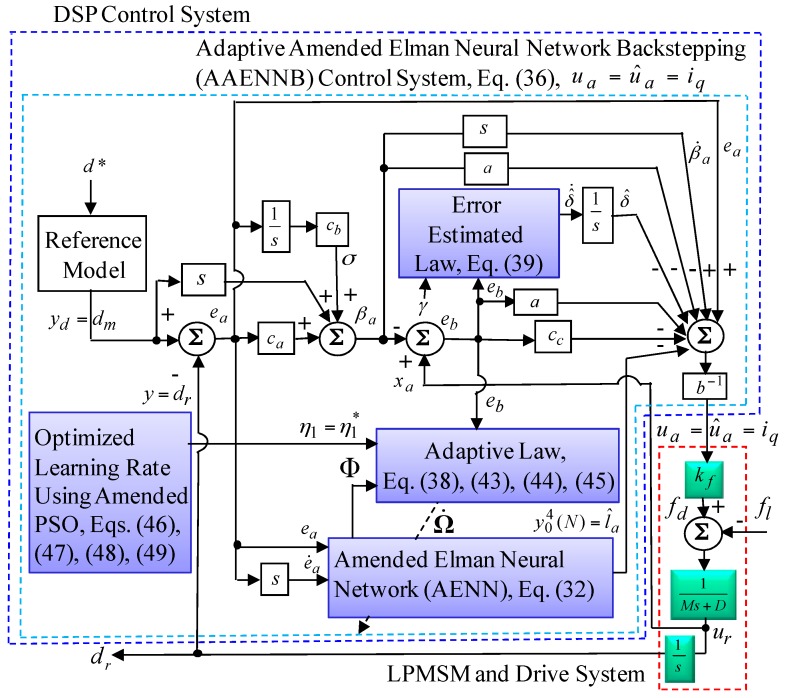
Block diagram of the proposed AAENNB control system using DSP control system.

By use of Barbalat’s lemma [38,39], it presented −ε(t)→0 as t→∞ from (26) and (27), then $e_{a}$ and $e_{b}$ will converge to zero as t→∞. Consequently, the stability of the proposed AAENNB control system can be guaranteed, and the control diagram block of the proposed AAENNB control system is shown in Figure 5. The persistent excitation condition [38,39] will be satisfied for the estimated value to converge to its theoretic value.

In order to train the AENN effectively, an on-line parameter training methodology can be derived by use of the adaptive law $\dot{\Omega }$ in (38). Then the adaptive law of the parameters in the AENN, $\dot{\Omega }(\mu_{jo},\mu_{kj},\mu_{ij},\mu_{oi})$ can be counted by use of the gradient descent method and the backpropagation algorithm, and these updated weights are presented in the following procedures.

The connective weight $\mu_{jo}$ can be updated by

(41) $${\dot{u}}_{jo}^{}=\eta_{1}e_{b}\Phi ≜-\eta_{1}\frac{\partial f_{b}}{\partial y_{o}^{4}}\frac{\partial y_{o}^{4}}{\partial net_{o}^{4}}\frac{\partial net_{o}^{4}}{\partial u_{jo}^{}}=-\eta_{1}\frac{\partial f_{b}}{\partial y_{o}^{4}}x_{j}^{4}$$

The above Jacobian term of controlled system can be presented as $\partial f_{b}/\partial y_{o}^{4}=-e_{b}$. The error term can be counted by

(42) $$\rho_{k}^{}≜-\frac{\partial f_{b}}{\partial y_{o}^{4}}=e_{b}$$

The connective weight $\mu_{kj}$ can be updated by

(43) $${\dot{u}}_{kj}^{}=-\frac{\partial f_{b}}{\partial u_{kj}}=-\frac{\partial f_{b}}{\partial y_{o}^{4}}\frac{\partial y_{o}^{4}}{\partial net_{o}^{4}}\frac{\partial net_{o}^{4}}{\partial y_{j}^{2}}\frac{\partial y_{j}^{2}}{\partial net_{j}^{2}}\frac{\partial net_{j}^{2}}{\partial u_{kj}}=\rho_{k}\mu_{jo}P_{j}$$

where $P_{j}\equiv \partial y_{j}^{2}/\partial u_{kj}$ can be counted from (29). 

The connective weight $\mu_{ij}$ can be updated by

(44) $${\dot{u}}_{ij}^{}=-\frac{\partial f_{b}}{\partial u_{ij}}=-\frac{\partial f_{2}}{\partial y_{o}^{4}}\frac{\partial y_{o}^{4}}{\partial net_{o}^{4}}\frac{\partial net_{o}^{4}}{\partial y_{j}^{2}}\frac{\partial y_{j}^{2}}{\partial net_{j}^{2}}\frac{\partial net_{j}^{2}}{\partial u_{ij}}=\rho_{k}\mu_{jo}Q_{j}$$

where $Q_{j}\equiv \partial y_{j}^{2}/\mu_{ij}$ can be counted from (29). 

The recurrent weight $\mu_{oi}$ can be updated by

(45) $${\dot{u}}_{oi}^{}=-\frac{\partial f_{2}}{\partial u_{oi}}=-\frac{\partial f_{2}}{\partial y_{o}^{4}}\frac{\partial y_{o}^{4}}{\partial y_{j}^{2}}\frac{\partial y_{j}^{2}}{\partial y_{i}^{1}}\frac{\partial y_{i}^{1}}{\partial u_{oi}}=\rho_{k}\mu_{jo}R_{j}$$

where $R_{j}\equiv \partial y_{j}^{2}/u_{oi}$ can be counted from (28). 

**Figure 6:**
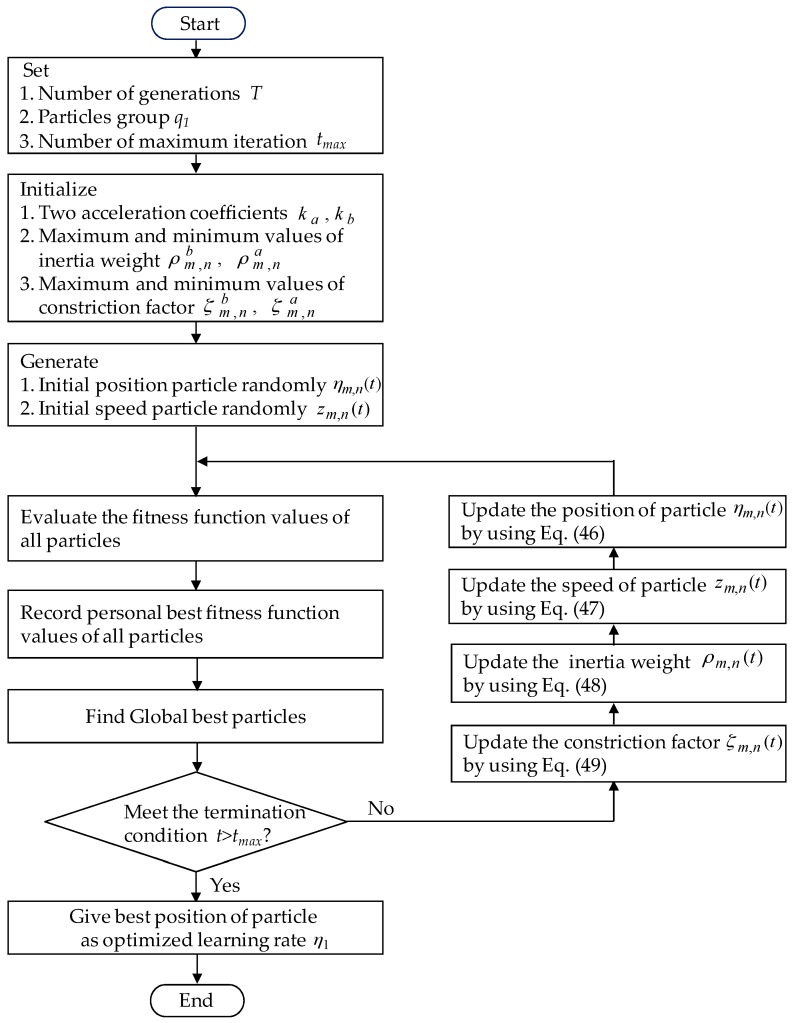
The flowchart result of an optimized learning rate using the mended PSO algorithm.

In order to acquire a better learning rate, the mended PSO is thus posed for finding the optimized learning rate of the weight in the AENN. Two acceleration coefficients $k_{a}$, $k_{b}$ and inertia weight $\rho_{m,n}(t)$ in the PSO [31,32,33,34,35] can result in an important effect on performance of the algorithm. Smaller inertia weight in the PSO results in a faster convergence speed and works well in local search. Larger inertia weight in the PSO can achieve a more accurate value and works well in global search. The dynamic modification of inertia weight is adopted for training the appropriate value of $\rho_{m,n}(t)$ in order to consort between find accuracy and find speed. Therefore, in order to speed up convergence, the mended PSO algorithm [36,37] is given by

(46) $$\eta_{m,n}(t+1)=\eta_{m,n}(t)+z_{m,n}(t+1),\;m=1,\;n=1,\;2,\;\ldots ,\;q_{1}$$

(47) $$z_{m,n}(t+1)=\rho_{m,n}(t)\;z_{m,n}(t)+\varsigma_{m,n}(t)k_{a}\phi_{m,n}^{a}(P_{m,n}^{b}-\eta_{m,n}(t))+\varsigma_{m,n}(t)k_{b}\phi_{m,n}^{b}(P_{m,n}^{g}-\eta_{m,n}(t)),\;m=1,n=1,\;2,\;\ldots ,\;q_{1}$$

(48) $$\rho_{m,n}(t)=\{\begin{array}{c} \rho_{m,n}^{a}+\frac{\phi_{m,n}^{c}(\rho_{m,n}^{a}-\rho_{m,n}^{b})(y_{m,n}-y_{m,n}^{a})}{\left(y_{m,n}^{c}-y_{m,n}^{a}\right)},\;if\;y\leq y_{m,n}^{a}\\ \rho_{m,n}^{b},\;if\;y>y_{m,n}^{c} \end{array},\;m=1,\;n=1,\;2,\;\ldots ,\;q_{1}$$

(49) $$\varsigma_{m,n}(t)=\varsigma_{m,n}^{a}+t\cdot \varsigma_{m,n}^{b}/T,\;m=1,\;n=1,\;2,\;\ldots ,q_{1}$$

where $\eta_{m,n}(t)$ is the current position of particle $P_{m,n}$ in the *n*th hyperspace at step *t* and with regard to an optimized learning rate $\eta_{m}^{*}(t),\;m=1$ at step *t*. $z_{m,n}(t)$ is the current speed of particle $P_{m,n}$ in the *n*th hyperspace at step *t*. $\rho_{m,n}(t)$ is the inertia weight within 0.4 < $\rho_{m,n}(t)$ < 0.9 [33,34] in the *n*th hyperspace at step *t*, so that search space can be changed steadily from global to local. $\rho_{m,n}^{b}$ and $\rho_{m,n}^{a}$ represent the maximum value and minimum value of $\rho_{m,n}(t)$ in the *n*th hyperspace. $\phi_{m,n}^{a}$, $\phi_{m,n}^{b}$ and $\phi_{m,n}^{c}$ are random numbers obtained from the uniform random distribution function in the interval [0, 1] in the *n*th hyperspace.$P_{m,n}^{b}$ and $P_{m,n}^{g}$ represent the best previous position of the *n*th hyperspace and the position of the best particle among all particles in the population in the *n*th hyperspace, respectively. $y_{m,n}$ is the current objective function value of particles. $y_{m,n}^{c}$, $y_{m,n}^{b}$ and $y_{m,n}^{a}$ are the average objective function value, the maximum objective function value and the minimum objective function value of all the current particles. $\varsigma_{m,n}^{a}$ and $\varsigma_{m,n}^{b}$ are the initial positive constants in the interval [0, 1] in the *n*th hyperspace. $t=1,2,\cdots t_{\max}$ denotes the number of the iteration. $t_{\max}$ denotes the number of the maximum iteration. *T* denotes the number of generations. $\varsigma_{m,n}(t)$ is the constriction factor [36,37] to avoid the swarm from premature convergence and to ensure stability of the system. In summary, the online tuning algorithm of the AENN is based on the adaptive law (38) for the connective weight adjustment by using the optimized learning rates $\eta_{m,n}^{}(t)=\eta_{m}^{*}(t),\;m=1$ in (41). Moreover, the AENN weight estimation errors are basically bounded [40]. The AENN weight estimation errors are bounded to ensure that the control signal is bounded. The flowchart which is shown Figure 6 presents the executed procedure of an optimized learning rate by using the mended PSO algorithm. 

## 3. Experimental Results and Discussion

**Figure 7:**
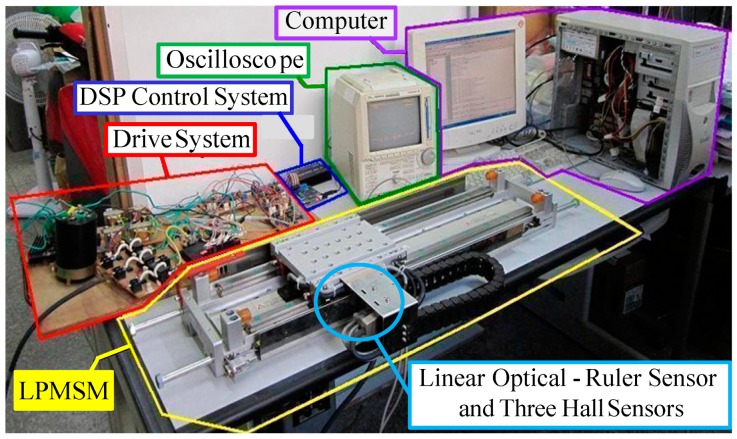
An experimental set-up picture of the LPMSM drive system.

The block diagram of the LPMSM drive system with a linear optical-ruler sensor and three Hall sensors by use of the DSP control system is presented in Figure 1. An experimental set-up picture of the LPMSM drive system is shown in Figure 7. The used DSP control system includes four sets of D/A converters, two sets of encoder interface circuits and eight sets of 16-bits input/output ports. The coordinate transformation in the field-oriented mechanism is enforced by the DSP control system. First, a 2nd-order transfer function with a rise time of 0.1 s is chosen as the reference model for the periodical step command [29]:

(50) $${\frac{d_{m}(s)}{d_{}^{*}(s)}|}_{f_{l}(s)=0}=\frac{1156}{s^{2}+68s+1156}$$

The control aim is to move the mover position to 4.0 mm periodically. Then, when the command is a sinusoidal reference trajectory, the reference model is set to be unit gain. The sampling interval of the control program in the experiment is set at 2 ms.

Five cases in the experimentat are offered in order to compare control performance by use of the eminent PI controller, the posed backstepping control system and the AAENNB control system. Case 1 is the nominal cases due to periodic step commands. Case 2 is the parameter disturbance with four times the nominal value of the mover mass and viscous friction due to periodic step commands. Case 3 is the nominal case due to periodic sinusoidal commands. Case 4 is the parameter disturbance with four times the nominal value of the mover mass and viscous friction due to periodic sinusoidal commands. Case 5 is the step force disturbance with adding load force as $f_{l}=2N$ via the opposite spring force. 

**Figure 8:**
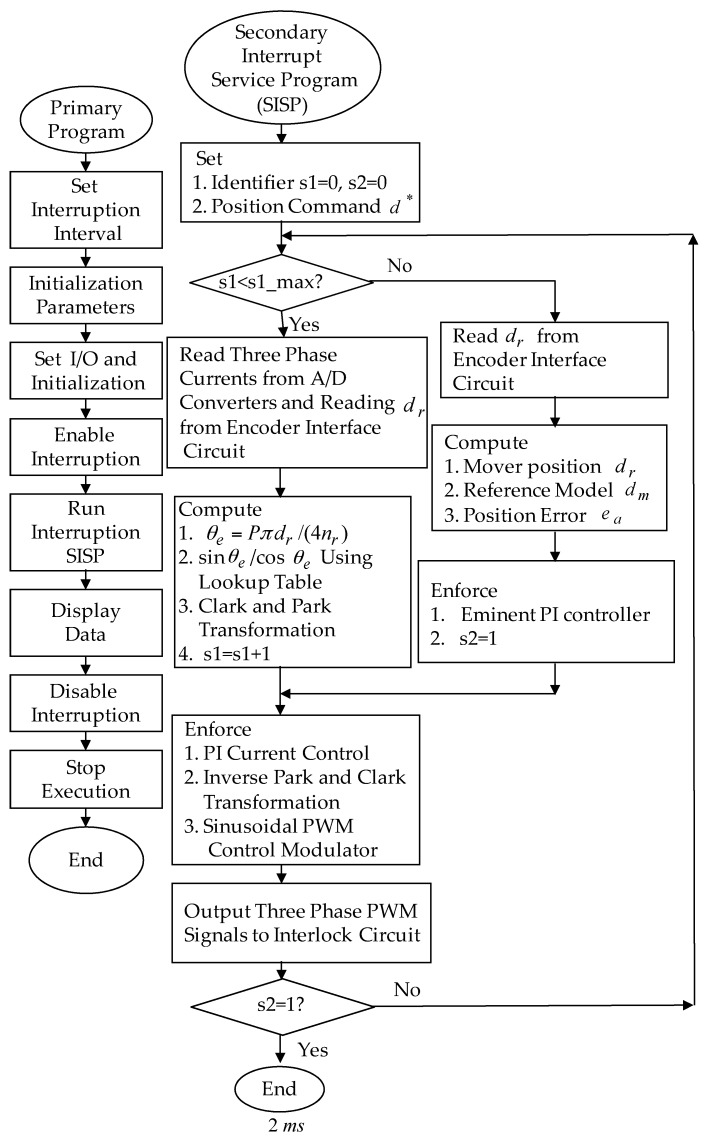
Flowchart of the enforced eminent PI controller program by use of the DSP control system.

**Figure 9:**
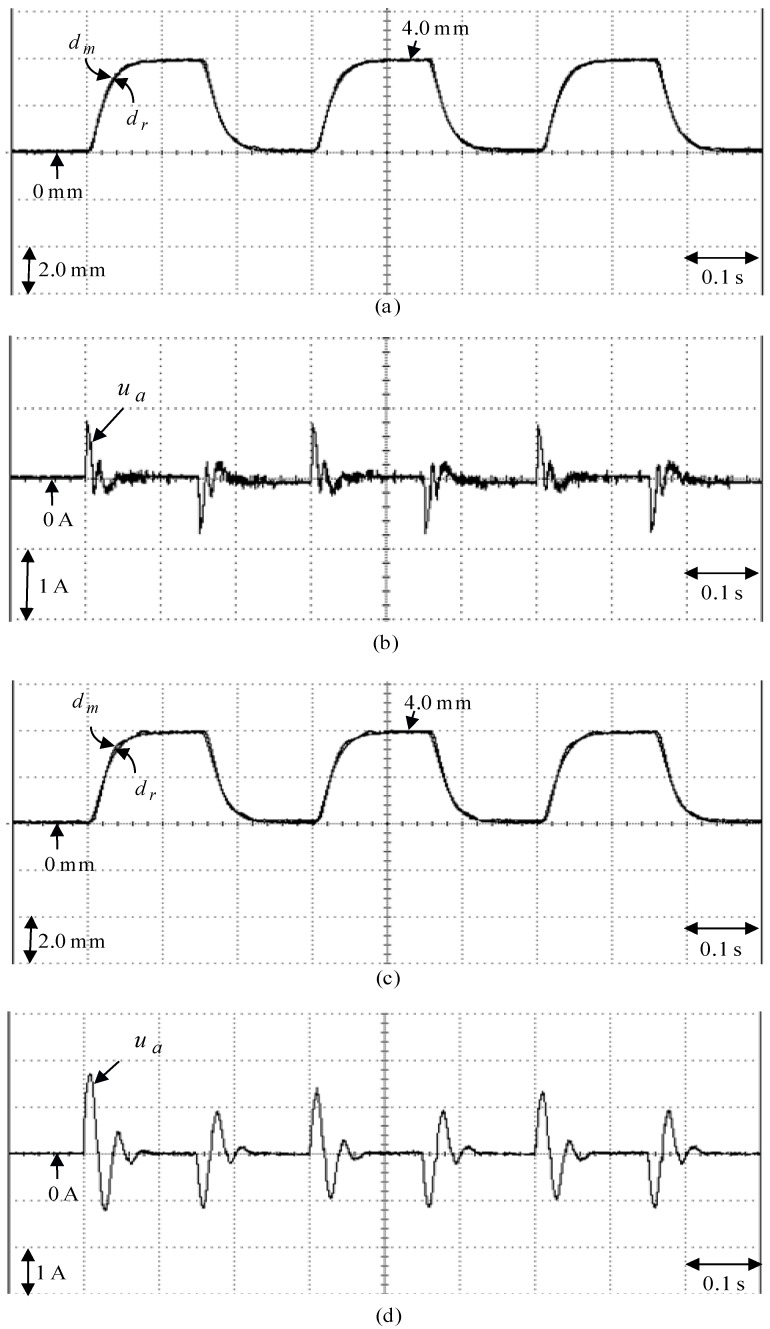
Experimental results of the eminent PI controller due to periodic step command from 0 mm to 4.0 mm: (**a**) position reaction of the mover in Case 1, (**b**) reaction of control effort in Case 1, (**c**) position reaction of the mover in Case 2, (**d**) reaction of control effort in Case 2.

**Figure 10:**
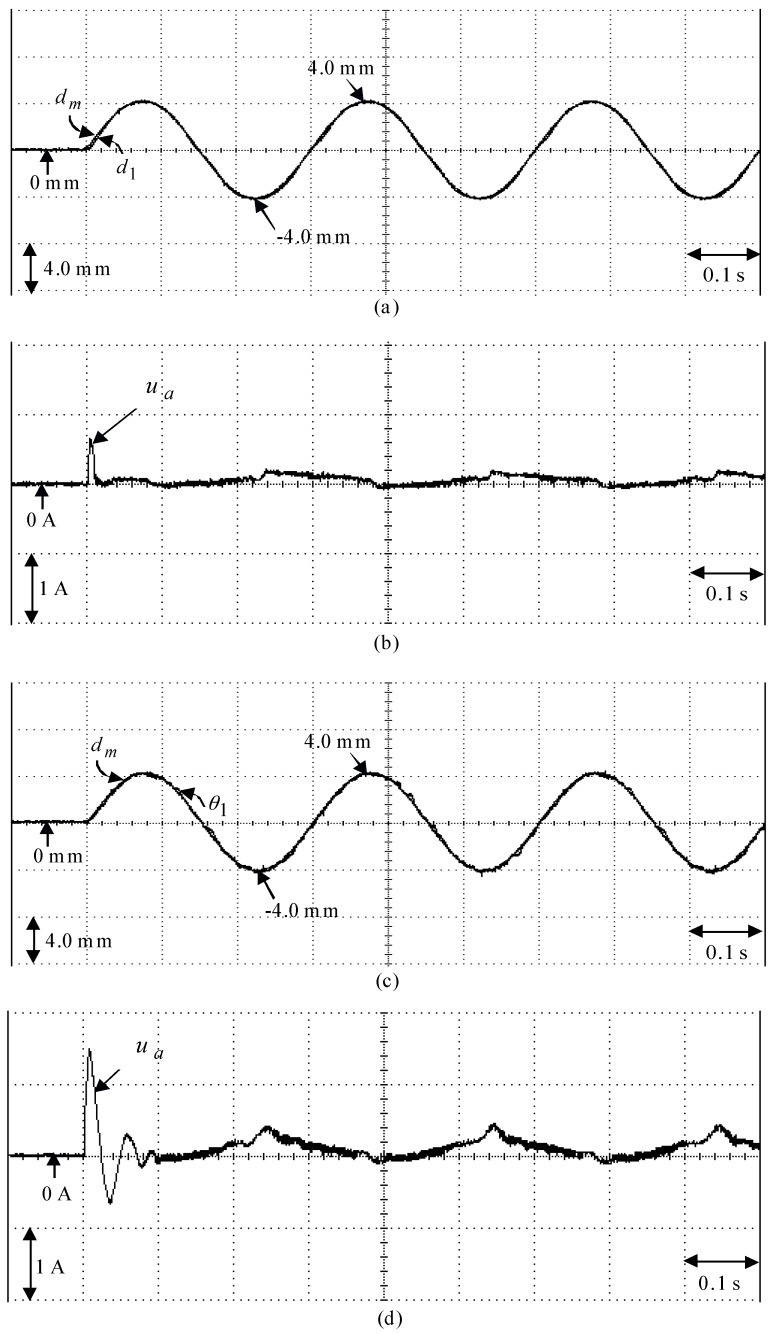
Experimental results of the eminent PI controller due to periodic sinusoidal command from −4.0 mm to 4.0 mm: (**a**) position reaction of the mover in Case 3, (**b**) reaction of control effort in Case 3, (**c**) position reaction of the mover in Case 4, (**d**) reaction of control effort in Case 4.

The eminent PI controller for the real-time control fulfillment in the DSP processors are comprised of the primary program and the secondary interrupt service program (SISP) in the DSP control system as illustrated in Figure 8. In the primary program, input/output (I/O) setting and parameters initialization are treated first. Then, the interrupt interval for the SISP is to be enabled. After enabling the interruption, the primary program is used to execute supervised control data. The important procedure of SISP with 2 ms sampling interval is to reailize for reading the mover position of the LPMSM drive system from a linear optical ruler sensor and three Hall sensors, reading three-phase currents from A/D converter, computing reference model and position error, enforcing lookup table and coordinate transformation, enforcing the eminent PI controller, and outputting three-phase current commands to switch the pulse-width-modulation (PWM) voltage source inverter with three sets of IGBT power modules by way of the isolated and delay-time circuits. The PWM voltage source inverter with three sets of insulated-gate-bipolar-transistor (IGBT) power modules is enforced by a ramp-comparison current-controlled PWM with a switching frequency of 15 kHz. Additionally, the measured bandwidth of the position loop control is about 80 Hz and the measured bandwidth of the current loop control is about 800 Hz for the LPMSM drive system by no-load test. The used controllers are all enforced by the DSP control system. The coordinate transformation in the field-oriented mechanism is also enforced by the DSP control system. To attain good transient-state and steady-state control performance, two gains of the eminent PI controller are $k_{pp}=4.1,$ and $k_{ip}=k_{pp}/T_{ip}=1.8$ by using the Kronecker method to construct a stability boundary in the $k_{pp}$ and $k_{ip}$ plane. This method is used to narrow down the region for iterative selection of values of the parameters of $k_{pp}$ and $k_{ip}$ [41,42,43] on the tuning of the PI controller in the nominal case for the position tracking. Figure 9 shows the experimental results of the eminent PI controller for controlling the LPMSM drive system due to periodic step command from 0 mm to 4 mm in the nominal case and in the parameter disturbance case as in Case 1 and Case 2. The position reactions of the mover in Case 1 and Case 2 due to periodic step command from 0 mm to 4 mm are illustrated in Figure 9a,c, respectively. The reactions of the associated control efforts with respect to Case 1 and Case 2 are illustrated in Figure 9b and d, respectively. Figure 10 is the experimental results of the eminent PI controller for controlling the LPMSM drive system in the nominal and parameter disturbance cases due to periodic sinusoidal command from −4 mm to 4 mm as in Case 3 and Case 4. The position reactions of the mover in Case 3 and Case 4 are shown in Figure 10a,c, respectively. The reactions of the associated control efforts with respect to Case 3 and Case 4 are shown in Figure 10b and d, respectively. The favorable tracing reactions of the position can be obtained by using the eminent PI controller in Case 1 and Case 3, as shown in Figure 9a and Figure 10a. Moreover, worse tracing reactions of position in Case 2 and Case 4, as shown in Figure 9c and Figure 10c, are very obvious due to the bigger nonlinear disturbance. From these experimental results, sluggish tracing reactions of position are obtained for controlling the LPMSM drive system by use of the eminent PI controller. Because of inappropriate rgulating two gains, the linear controller has weak robustness under the bigger nonlinear disturbance.

**Figure 11:**
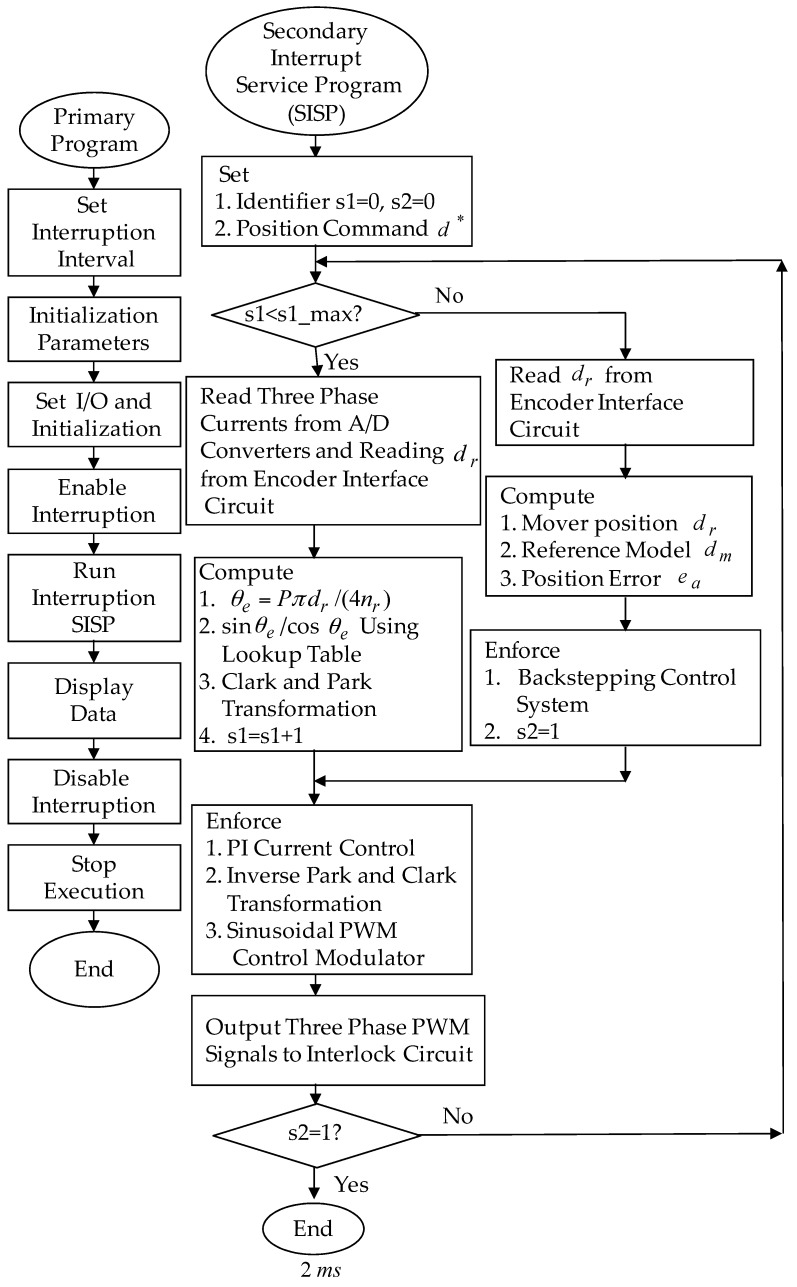
Flowchart of the enforced posed backstepping control system program by use of DSP control system.

**Figure 12:**
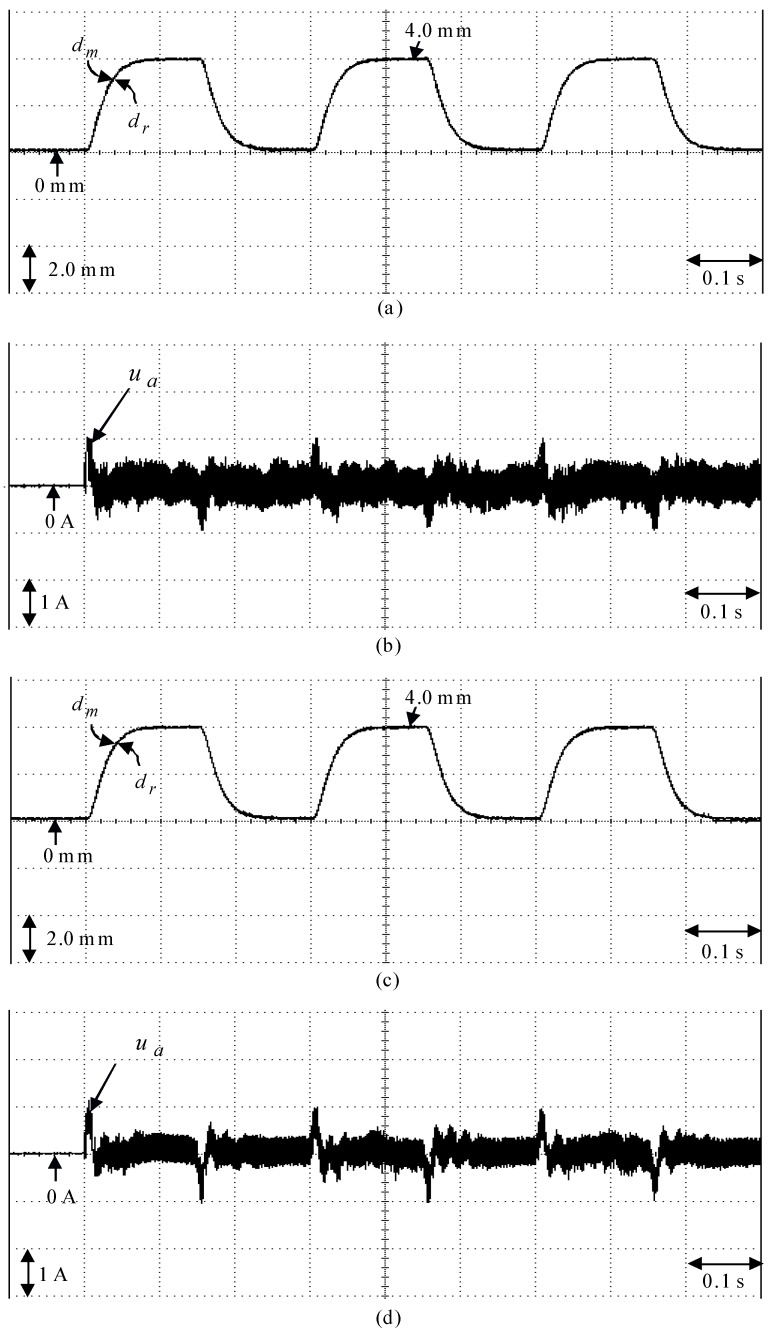
Experimental results of the posed backstepping control system due to periodic step command from 0 mm to 4.0 mm: (**a**) position reaction of the mover in Case 1, (**b**) reaction of control effort in Case 1, (**c**) position reaction of the mover in Case 2, (**d**) reaction of control effort in Case 2.

**Figure 13:**
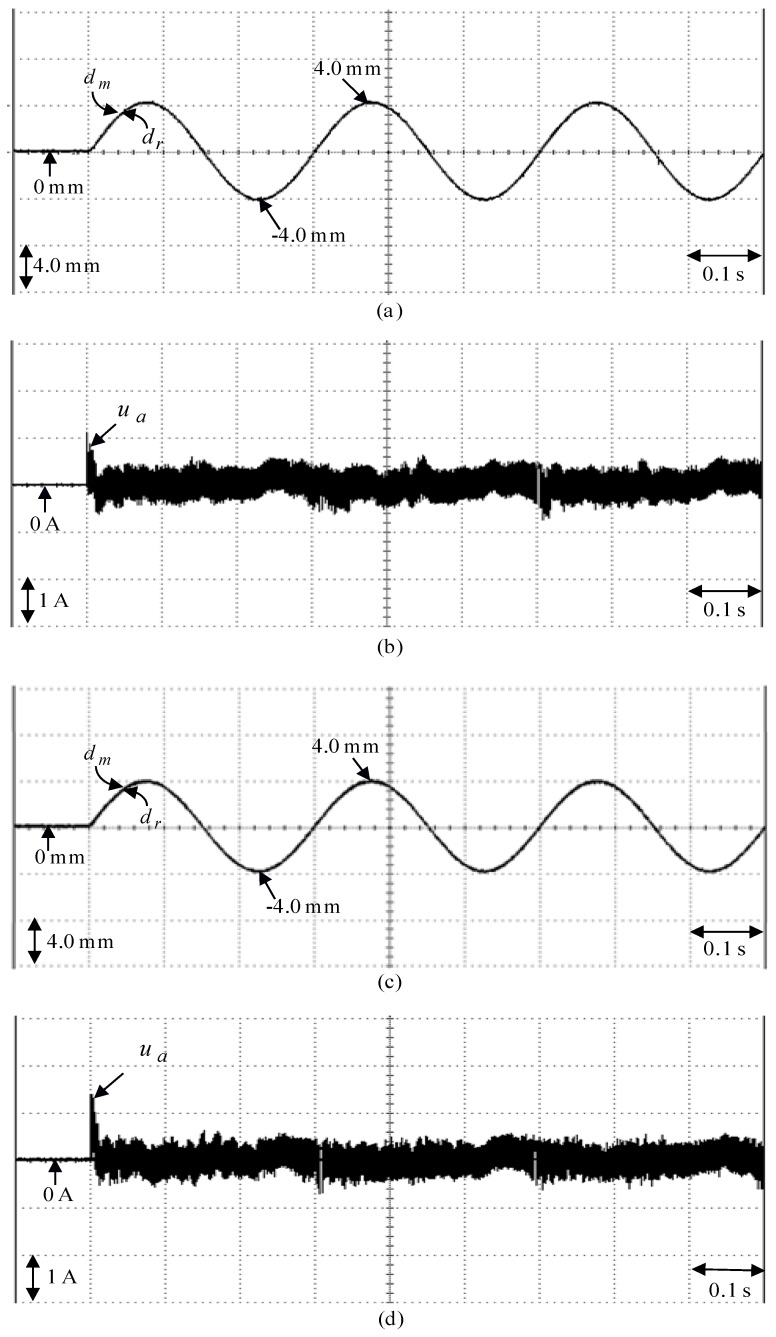
Experimental results of the posed backstepping control system due to periodic sinusoidal command from −4.0 mm to 4.0 mm: (**a**) position reaction of the mover in Case 3, (**b**) reaction of control effort in Case 3, (**c**) position reaction of the mover in Case 4, (**d**) reaction of control effort in Case 4.

The posed backstepping control system for the real-time control fulfillment in the DSP processors are comprised of the primary program and the secondary interrupt service program (SISP) in the DSP control system as illustrated in Figure 11. In the primary program, input/output (I/O) setting and parameters initialization are treated first. Then, the interrupt interval for the SISP is to be enabled. After enabling the interruption, the primary program is used to execute supervised control data. The important procedure of SISP with 2 ms sampling interval is to reailize for reading the mover position of the LPMSM drive system from a linear optical ruler sensor and three Hall sensors, reading three-phase currents from A/D converter, computing reference model and position error, enforcing lookup table and coordinate transformation, enforcing the posed backstepping control system, and outputting three-phase current commands to switch the pulse-width-modulation (PWM) voltage source inverter with three sets of IGBT power modules by way of the isolated and delay-time circuits. The PWM voltage source inverter with three sets of insulated-gate-bipolar-transistor (IGBT) power modules is enforced by a ramp-comparison current-controlled PWM with a switching frequency of 15 kHz. Additionally, the measured bandwidth of position loop control is about 80 Hz and the measured bandwidth of current loop control is about 800 Hz for the LPMSM drive system by no-load test. The used controllers are all enforced by the DSP control system. The coordinate transformation in the field-oriented mechanism is also enforced by the DSP control system. Four parameters of the posed backstepping control system are given as $c_{a}=2.4$, $c_{b}=2.5$, $c_{c}=2.3$ and ${\bar{l}}_{a}=8.2$ according to heuristic lore [4,5], resulting in the periodic step command from 0 mm to 4.0 mm in the nominal case for the position tracing to achieve good transient-state and steady-state control performance. Figure 12 is the experimental result of the posed backstepping control for controlling the LPMSM drive system due to periodic step command from 0 mm to 4.0 mm in the nominal case and in the parameter disturbance case as in Case 1 and Case 2. Figure 13 is the experimental result of the posed backstepping control system for controlling the LPMSM drive system due to periodic sinusoidal command from −4.0 mm to 4.0 mm in the nominal case and in the parameter disturbance case as in Case 3 and Case 4. The position reactions of the mover in Case 1 and Case 2 are shown in Figure 12a,c, respectively. The reactions of the associated control efforts with respect to Case 1 and Case 2 are shown in Figure 12b,d, respectively. The position reactions of the mover in Case 3 and Case 4 are shown in Figure 13a,c, respectively. The reactions of the associated control efforts with respect to Case 3 and Case 4 are shown in Figure 13b,d, respectively. The favorable tracing reactions of position can be obtained by the use of the posed backstepping control system in Case 1 and Case 3, as shown in Figure 12a and Figure 13a. Meanwhile, fine tracing reactions of position in Case 2 and Case 4, as shown in Figure 12c and Figure 13c, are obvious under the bigger nonlinear disturbance. From these experimental results, good tracing reactions of position are obtained for controlling the LPMSM drive system by use of the posed backstepping control system in Case 1, Case 2, Case 3 and Case 4. However, the larger upper bound with the switching function in the control effort caused serious vibration. Moreover, the vibration of control effort will wear the bearing mechanism and might excite unstable system dynamics.

**Figure 14:**
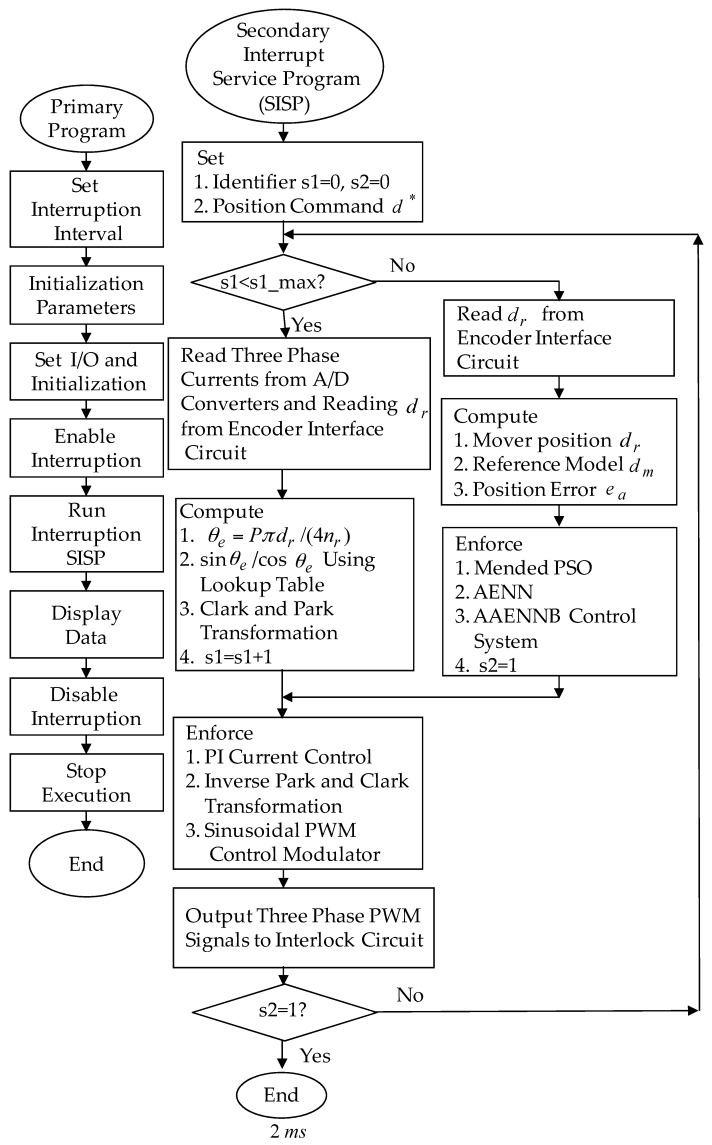
Flowchart of the enforced proposed AAENNB control system program by use of DSP control system.

**Figure 15:**
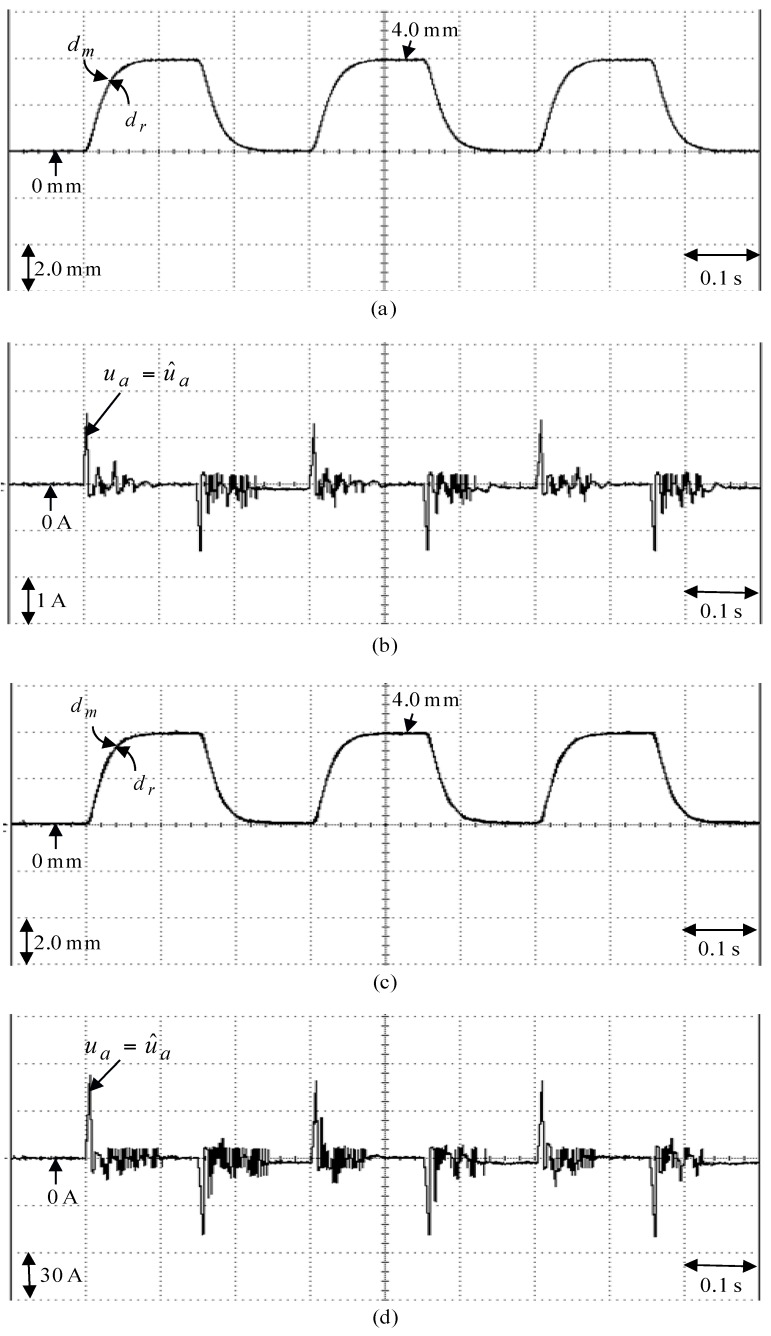
Experimental results of the proposed AAENNB control system due to periodic step command from 0 mm to 4.0 mm: (**a**) position reaction of the mover in Case 1, (**b**) reaction of control effort in Case 1, (**c**) position reaction of the mover in Case 2, (**d**) reaction of control effort in Case 2.

**Figure 16:**
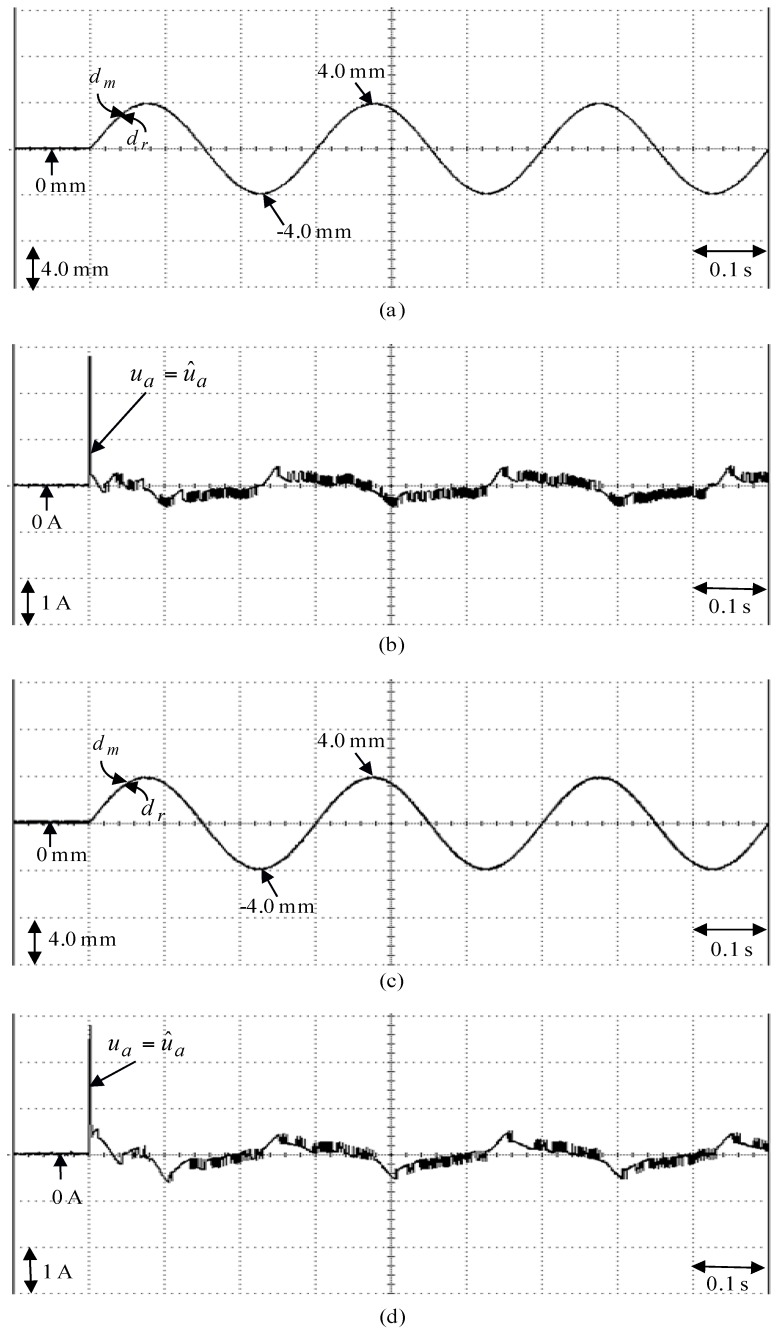
Experimental results of the proposed AAENNB control system due to periodic sinusoidal command from −4.0 mm to 4.0 mm: (**a**) position reaction of the mover in Case 3, (**b**) reaction of control effort in Case 3, (**c**) position reaction of the mover in Case 4, (**d**) reaction of control effort in Case 4.

The proposed AAENNB control system for the real-time control fulfillment in the DSP processors are comprised of the primary program and the secondary interrupt service program (SISP) in the DSP control system, as illustrated in Figure 14. In the primary program, input/output (I/O) setting and parameters initialization are treated first. Then, the interrupt interval for the SISP is to be enabled. After enabling the interrupt, the primary program is used to execute supervised control data. The important procedure of SISP with 2 ms sampling interval is to reailize for reading the mover position of the LPMSM drive system from a linear optical ruler sensor and three Hall sensors, reading three-phase currents from A/D converter, computing reference model and position error, enforcing lookup table and coordinate transformation, enforcing the proposed AAENNB control system, and outputting three-phase current commands to the switch pulse-width-modulation (PWM) voltage source inverter with three sets of IGBT power modules by way of the isolated and delay-time circuits. The PWM voltage source inverter with three sets of insulated-gate-bipolar-transistor (IGBT) power modules is enforced by a ramp-comparison current-controlled PWM with a switching frequency of 15 kHz. Additionally, the measured bandwidth of the position loop control is about 80 Hz and the measured bandwidth of the current loop control is about 800 Hz for the LPMSM drive system by no-load test. The used controllers are all enforced by the DSP control system. The coordinate transformation in the field-oriented mechanism is also enforced by the DSP control system. To show the effectiveness of the control system with a small number of neurons, the AENN has 2, 6, 6 and 1 neurons in the input, hidden, context and output layers, respectively, because of providing fast convergence and better transient-state and steady-state responses. Five parameters of the proposed AAENNB control system: $c_{a}=2.4$, $c_{b}=2.5$, $c_{c}=2.3$, γ=0.1 and α=0.2 through some heuristic lore [4,5], [28,29,30] result in the periodic step command from 0 mm to 4.0 mm in the nominal case for the position tracing in order to achieve good transient-state and steady-state control performance. The parameter adjustment process remains continually active for the duration of the experimentation. The parameter’s initialization of the AENN in Reference [40] is adopted to initialize the parameters in this paper. Figure 15 is the experimental result of the proposed AAENNB control system for controlling the LPMSM drive system due to periodic step command from 0 mm to 4.0 mm in the nominal case and in the parameter disturbance case as in Case 1 and Case 2. Figure 16 is the experimental result of the proposed AAENNB control system for controlling the LPMSM drive system due to periodic sinusoidal command from −4.0 mm to 4.0 mm in the nominal case and in the parameter disturbance case as in Case 3 and Case 4. The position reactions of the mover in Case 1 and Case 2 are shown in Figure 15a,c, respectively. The reactions of the associated control efforts with respect to Case 1 and Case 2 are shown in Figure 15b,d, respectively. The position reactions of the mover in Case 3 and Case 4 are shown in Figure 16a,c, respectively. The reactions of the associated control efforts with respect to Case 3 and Case 4 are shown in Figure 16b,d, respectively. The best tracing reactions of position can be obtained by use of the proposed AAENNB in Case 1 and Case 3, as shown in Figure 15a and Figure 16a. Moreover, excellent tracing reactions of position in Case 2 and Case 4 are shown in Figure 15c and 16c and are very conspicuous under the bigger nonlinear disturbance. From these experimental results, better tracking reactions of position are obtained by the use of the proposed AAENNB control system for controlling the LPMSM drive system. Moreover, the vibration of control efforts in Case 1, Case 2, Case 3 and Case 4 are much reduced by the use of the AAENNB control system as shown in Figure 15b,d, and Figure 16b,d, respectively. However, the robust control performances of the proposed AAENNB control system under the occurrence of parameter variations in the different trajectories are obvious owing to the on-line adaptive adjustment of the AENN. From the experimental results, the control performance of the proposed AAENNB control system is better than the control performance of the backstepping control system for the tracing of periodical commands. 

**Figure 17:**
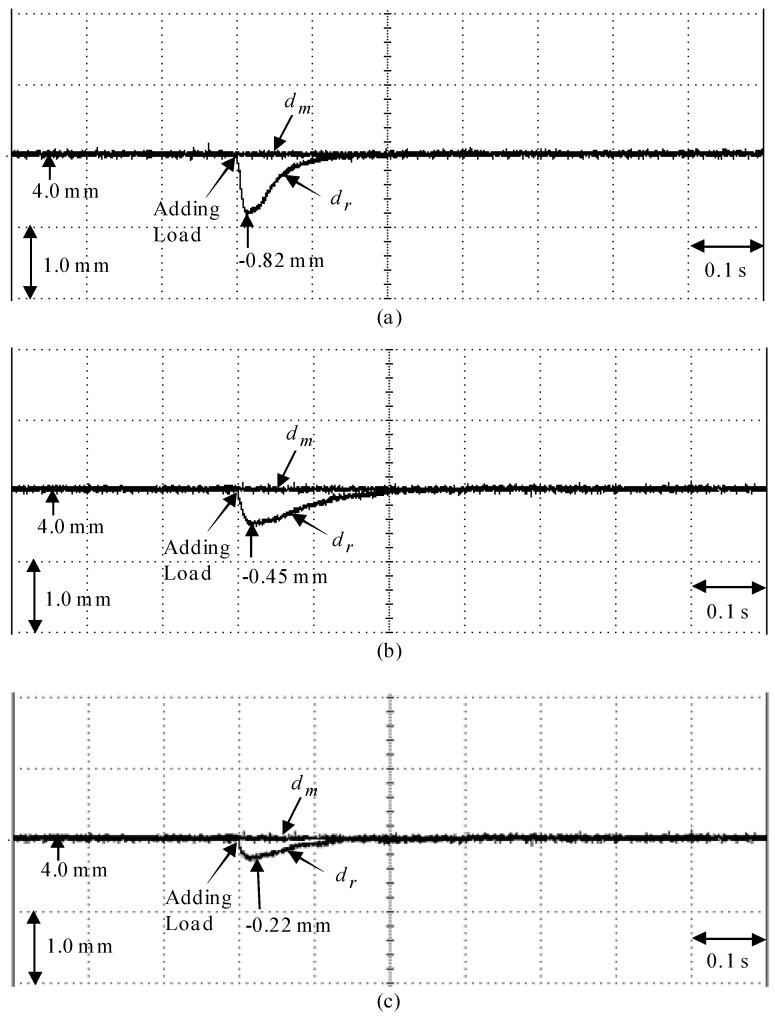
Experimental results of measured mover position reaction in Case 5: (**a**) by use of the renowned PI controller, (**b**) by use of the backstepping control system, (**c**) by use of the proposed AAENNB control system.

Finally, experimental results of the measured mover position reaction under step disturbance torque with adding load force as $f_{l}=2N$ via the opposite spring force at 4 mm, i.e., Case 5, is illustrated in Figure 17 by use of the eminent PI controller, the posed backstepping control system, and the proposed AAENNB control system. Experimental results of the measured mover position reaction by use of the eminent PI controller in Case 5 is illustrated in Figure 17a. Experimental results of the measured mover position reaction by use of the posed backstepping control system in Case 5 is illustrated in Figure 17b. Experimental results of the measured mover position reaction by use of the proposed AAENNB control system in Case 5 is illustrated in Figure 17c. From these experimental results, the transient reaction of the proposed AAENNB control system is better than the eminent PI controller and the posed backstepping control system at load force regulation. However, the robust control performance of the proposed AAENNB control system was outstanding for controlling the LPMSM drive system in the tracing of periodic step and sinusoidal commands under the occurrence of parameter disturbance, and the load force regulation owing to the on-line adaptive adjustment of the AENN.

Additionally, the comparison of the control performances of the eminent PI controller, the posed backstepping control system, and the proposed AAENNB control system is enumerated in Table 1 with respect to the experimental results of five test cases. The maximum errors of $e_{a}$ by use of the eminent PI controller, the posed backstepping control system and the proposed AAENNB control system in Case 1 are 0.64 mm, 0.35 mm and 0.19 mm, respectively. The root-mean-square (RMS) errors of $e_{a}$ by use of the eminent PI controller, the posed backstepping control system and the proposed AAENNB control system in Case 1 are 0.45 mm, 0.21 mm and 0.08 mm, respectively. The maximum errors of $e_{a}$ by use of the eminent PI controller, the posed backstepping control system and the proposed AAENNB control system in Case 2 are 0.82 mm, 0.43 mm and 0.23 mm, respectively. The RMS errors of $e_{a}$ by use of the eminent PI controller, the posed backstepping control system and the proposed AAENNB control system in Case 2 are 0.51 mm, 0.25 mm and 0.09 mm, respectively. The maximum errors of $e_{a}$ by use of the eminent PI controller, the posed backstepping control system and the proposed AAENNB control system in Case 3 are 0.63 mm, 0.34 mm and 0.18 mm, respectively. The RMS errors of $e_{a}$ by use of the eminent PI controller, the posed backstepping control system and the proposed AAENNB control system in Case 3 are 0.38 mm, 0.19 mm and 0.07 mm, respectively. The maximum errors of $e_{a}$ by use of the eminent PI controller, the posed backstepping control system and the proposed AAENNB control system in Case 4 are 0.81 mm, 0.44mm and 0.22 mm, respectively. The RMS errors of $e_{a}$ by use of the eminent PI controller, the posed backstepping control system and the proposed AAENNB control system in Case 4 are 0.48 mm, 0.23 mm and 0.09 mm, respectively. The maximum errors of $e_{a}$ by use of the eminent PI controller, the posed backstepping control system and the proposed AAENNB control system in Case 5 are 0.82 mm, 0.45 mm and 0.22 mm, respectively. The RMS errors of $e_{a}$ by use of the eminent PI controller, the posed backstepping control system and the proposed AAENNB control system in Case 5 are 0.54 mm, 0.28 mm and 0.10 mm, respectively. As a result, the proposed AAENNB control system has a smaller tracing error in comparison with the eminent PI controller and the posed backstepping control system from Table 1. According to the tabulated measurements, the proposed AAENNB control system indeed yields the better control performance. 

Furthermore, the characteristic performance comparisons of the eminent PI controller, the posed backstepping control system and the proposed AAENNB control system are enumerated in Table 2 for experimental results. In Table 2, the various performances with respect to the vibration of control effort, the dynamic response, the ability of load regulation, the convergence speed, the position tracing error, and the rejection ability of parameter disturbance in the proposed AAENNB control system are superior to the eminent PI controller and the posed backstepping control system. 

## 4. Conclusions

The LPMSM drive system with a linear optical-ruler sensor and three Hall sensors for the tracing of periodic reference inputs is controlled by the proposed AAENNB control system. The main contributions of this study are as follows: (1) The field-oriented mechanism has been successfully applied for controlling the LPMSM drive system with a linear optical-ruler sensor and three Hall sensors; (2) the posed backstepping control system has been successfully derived according to the Lyapunov function to reduce the influence under the lumped uncertainty disturbances; (3) the proposed AAENNB control system has been successfully derived according to the Lyapunov function for reducing the lumped uncertainty affect; (4) the AENN with adaptive law has been successfully estimated the lumped uncertainty; (5) the error estimated law has been successfully compensated the observed error induced by the AENN with adaptive law for diminishing the lumped uncertainty effect; (6) the mended PSO has been successfully applied for regulating the optimal learning rate of the AENN to raise convergent speed. 

Furthermore, as indicated by the experimental results in Table 1, the proposed AAENNB control system has a smaller tracing error and better disturbance rejection in comparison with the eminent PI controller and the posed backstepping control system. 

Finally, the comparisons of the various control performances are verified by the experimental results and the the proposed AAENNB control system is superior to those of the eminent PI controller and the posed backstepping control system with respect to the vibration of control effort, the dynamic response, the ability of load regulation, the convergent speed, the position tracing error, and the rejection ability of parameters disturbance.

## Figures and Tables

**Table 1:** Performance comparison of control systems.

Control System and Five Cases	Performance	Case 1	Case 2	Case 3	Case 4	Case 5
**Eminent PI controller**	**Maximum error of** $e_{a}$	0.64 mm	0.82 mm	0.63 mm	0.81 mm	0.82 mm
	**RMS error of** $e_{a}$	0.45 mm	0.51 mm	0.38 mm	0.48 mm	0.54 mm
**Backstepping control system**	**Maximum error of** $e_{a}$	0.35 mm	0.43 mm	0.34 mm	0.44 mm	0.45 mm
	**RMS error of** $e_{a}$	0.21 mm	0.25 mm	0.19 mm	0.23 mm	0.28 mm
**AAENNB control system**	**Maximum error of** $e_{a}$	0.19 mm	0.23 mm	0.18 mm	0.22 mm	0.22 mm
	**RMS error of** $e_{a}$	0.08 mm	0.09 mm	0.07 mm	0.09 mm	0.10 mm

**Table 2:** Characteristic performance comparisons of control systems.

Control System	Eminent PI Controller	Backstepping Control System	AAENNB Control System
Characteristic Performance			
**Vibration in control effort**	Smaller	Larger	Smaller
**Dynamic response**	Slower	Faster	Fastest
**Load regulation capability**	Poor (maximum error as 0.82 mm with adding load at 4.0 mm)	Good (maximum error as 0.45 mm with adding load at 4.0 mm)	Best (maximum error as 0.22 mm with adding load at 4.0 mm)
**Convergent speed**	Slower	Faster	Fastest
**Position tracing error**	Large	Middle	Small
**Rejection for parameters disturbance**	Poor	Good	Best
**Learning rate**	-	-	Vary (optimal rate)
